# Hyperbolic topological data analysis mapper reveals dynamic trait–environment patterns in plant phenomics

**DOI:** 10.1016/j.plaphe.2026.100186

**Published:** 2026-02-27

**Authors:** Jan Zdražil, Lingping Kong, Lukáš Spíchal, Václav Snášel, Nuria De Diego

**Affiliations:** a Czech Advanced Technology and Research Institute (CATRIN), Palacký University Olomouc, Slechtitelů 27, 77900, Olomouc, Czech Republic; b Faculty of Electrical Engineering and Computer Science, VSB-Technical University of Ostrava, Ostrava, Czech Republic; c Center for Advanced Technologies and Engineering (CATEN), Technologická 375/3, 708 00, Ostrava-Pustkovec, Czech Republic

**Keywords:** Plant phenomics, Euclidean geometry, Hyperbolic geometry, Contrasting learning, Data visualization

## Abstract

Modern plant phenotyping faces the challenge of interpreting complex, high-dimensional data. Traditional analytical tools often fail to capture the non-linear, hierarchical, and temporal relationships that define plant responses under multifactorial conditions. We present the Hyperbolic Topological Data Analysis Mapper (HTDA-Mapper), a novel algorithm designed to overcome these limitations by embedding data in Poincaré ball space. Unlike conventional Euclidean approaches, HTDA-Mapper preserves the hierarchical structure of phenotypic traits, improves cluster resolution, and reveals hidden growth trajectories across treatments and time, offering a powerful means to explore latent phenoms. The pipeline supports both quantitative data and images. When integrated with unsupervised contrastive learning, HTDA-Mapper identifies similarities and differences in raw image data without requiring manual labelling or post hoc processing. We applied this framework to a high-throughput phenotyping (HTP) dataset of over 27,000 images of *Arabidopsis thaliana* seedlings exposed to varying nutrient levels and priming agents at different concentrations over seven days. Using cubical complexes, HTDA-Mapper mapped relationships between treatment variables, compound concentrations, and phenotypic outcomes. Furthermore, it reliably detected compound-specific effects, uncovered dynamic trait–environment interactions, revealed phenotypic trajectories not captured by conventional methods, and facilitated biologically meaningful interpretation of the complex dataset. By preserving the geometry and temporal evolution of plant development, HTDA-Mapper sets a new standard for HTP analysis. Beyond phenomics, it is a versatile tool for other omics, such as transcriptomics and metabolomics, where structured, high-dimensional data is prevalent. HTDA-Mapper can accelerate data-driven crop improvement by uncovering effective compounds, robust genotypes, and adaptive growth strategies that enhance plant resilience.

## 1 Introduction

Plants live in environments that constantly challenge them, and, as sessile organisms, they cannot escape these conditions. Drought, heat, poor soil nutrients, and diseases all affect how plants grow and develop. As the global population increases and the climate continues to change, we urgently need to make agriculture more efficient and sustainable by reducing costs and minimising the environmental impacts with the development of new agrotechnologies [1], with a particular focus on new compounds and strategies that reduce the use of natural resources and enhance plant growth and stress tolerance.

One promising strategy is the use of plant growth regulators, compounds that help plants grow stronger or perform better under adverse conditions. However, finding the right compound, dose, and timing is a complex process. Each plant can respond differently to various environments and types of stress, making it challenging to test multiple factors simultaneously in traditional laboratory experiments. High-throughput phenotyping (HTP) offers a solution [2]. It uses automated imaging and sensors installed in growth chambers, tractors, or drones to measure thousands of plants quickly and without damaging them [[3], [4], [5]]. HTP systems, hence, track how plants grow and respond to stress over time, producing vast amounts of data, including images, manual measurements, and other observations. However, this big data also creates a new challenge: **how to analyse and interpret huge, complex datasets effectively** to reveal deeper, hidden relationships within them, a task in which traditional statistics cannot always help.

In plant phenomics, the most commonly used artificial intelligence (AI) tools are supervised learning models trained on data that humans have already labelled (e.g., marking each image as “healthy” or “stressed”). While powerful, these methods depend on large, carefully prepared datasets and can only recognise patterns that researchers already know to look for. Unsupervised learning can overcome these limitations by taking a different approach. Instead of relying on labels, it allows AI to explore the data freely and find patterns on its own. This is especially important in plant phenomics, where biological processes are complex, and many interactions among genes, environments, and growth conditions remain unknown.

Concretely, we developed the Hyperbolic Topological Data Analysis (HTDA)-Mapper, initially proposed by Ref. [6], an innovative approach that helps scientists visualise the shape of large plant datasets. HTDA-Mapper, which builds upon Mapper, a powerful tool from topological data analysis (TDA), is a mathematical approach that focuses on the intrinsic structures and connectivity of data rather than relying solely on raw numerical values. What makes HTDA-Mapper unique is the use of hyperbolic geometry, a space that better reflects hierarchical and nested structures compared to traditional Euclidean geometry [7]. Recent findings suggest that when a concept repeatedly appears across mathematical domains with substantial influence, it warrants independent investigation [8,9]. Accordingly, we propose an innovative approach to plant growth and development monitoring, grounded in manifold theory and leveraging hyperbolic geometry —a particularly effective model for data with hierarchical relationships. Specifically, in plant phenomics, where traits such as leaf development, branching, and stress responses often evolve in tree-like patterns, hyperbolic space helps preserve this complexity without distorting it. This results in more precise visualisations and a deeper understanding of how traits change over time under different conditions.

Unlike conventional Mapper methods, HTDA-Mapper utilises the Poincaré ball model and cubical complexes to represent hierarchical relationships in data more effectively. It can process not only numerical data but also raw images by integrating contrastive learning, a self-supervised AI technique that trains the model to recognise similarities and differences in raw images without human labelling. This ability to “learn by itself”, applying ML models such as the Simple Framework for Contrastive Learning of Visual Representations (SimCLR) [10] or Bootstrap Your Own Latent (BYOL) [11], enables our approach to end-to-end analysis of raw images and makes it well-suited for discovering new patterns hidden in complex biological data.

**Fig. 1:**
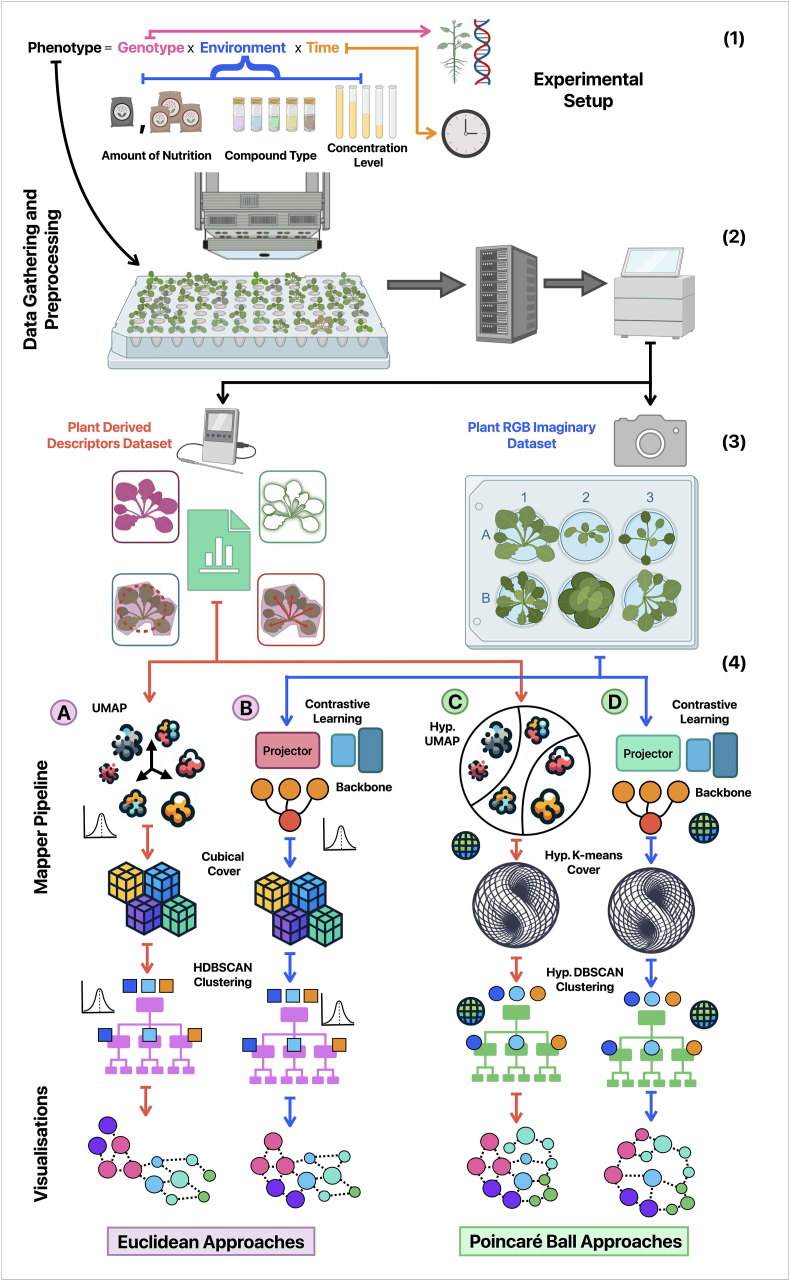
Comparison of the Euclidean *vs.* HTDA-Mapper Pipelines for *Arabidopsis thaliana* Data Analysis. This illustration shows the analytical pathways used for plant-derived descriptors and high-resolution image datasets separately in Euclidean and hyperbolic spaces. **(A)** Depicts the Euclidean mapper pipeline for plant-derived descriptors, starting with UMAP for feature reduction, proceeding through a cubical cover function, and finalising with clustering to generate the mapper graph. **(B)** Highlights the pathway for image datasets within Euclidean space, where embeddings generated via Euclidean contrastive learning techniques are segmented by a cubical cover and clustered to produce the mapper graph. The right side of the figure introduces the hyperbolic mapper strategy **(C)** for plant-derived descriptors, incorporating hyperbolic UMAP for feature reduction onto the Poincaré ball, followed by our proprietary Poincaré ball cover function and hyperbolic clustering to yield the mapper graph. **(D)** Illustrates the approach for image datasets in the hyperbolic space, where hyperbolic contrastive learning yields Poincaré disk embeddings, which are subsequently processed using the same cover function and clustering mechanism as those in (C). This comparison underscores the nuanced but significant distinctions and synergies between Euclidean and hyperbolic mapping methodologies in uncovering complex patterns and phenotypic variations within *Arabidopsis* datasets.

In this study, we applied HTDA-Mapper to more than 27,000 images of *Arabidopsis thaliana* seedlings grown under various nutrient and chemical treatments as a proof-of-concept to demonstrate the power of this new tool for analysing HTP data from multifactorial experiments (Fig. 1). Throughout this work and using this approach, we use the term “non-linear relationships” to refer to curved and branching progression patterns in trait or embedding space as plants evolve over time and under different treatments, and “hierarchical relationships” to refer to the multi-level organisation of plants into nested groups defined by their growth condition and time. In particular, the data exhibit a biologically meaningful coarse-to-fine organization that reflects how interventions affect plant growth. The nutrient regime represents the broadest environmental constraint, defining overall resource availability and shaping the baseline growth trajectory. Within each nutrient context, the priming compound constitutes a more specific perturbation, allowing us to assess whether the addition of a single agent can redirect development and whether closely related molecules induce distinct phenotypic responses. Finally, concentration acts at the finest scale, modulating the strength of a given compound's effect and capturing dose-dependent behaviour. This ordering (nutrient regime → compound → concentration) therefore reflects a biologically meaningful progression from global environmental limitation to molecular identity and, ultimately, effect intensity, and provides a consistent hierarchical framework for interpreting plant responses across all analyses in this study.

Our results show that our approach can reveal new growth patterns and interactions that standard methods often overlook. The pipeline was sensitive enough to detect subtle, concentration-dependent effects and similarities and dissimilarities between compounds, even among chemically related molecules. By combining two unsupervised ML methods, such as manifold learning with hyperbolic topology and contrastive learning, HTDA-Mapper provides a powerful and intuitive approach to explore plant behavior, i) visualising complex, high-dimensional plant traits related to the *latent phenom* more effectively, ii) identifying promising compounds and concentrations that enhance plant performance under concrete growth conditions, and iii) accelerating discovery in plant science by turning big data into actionable insights.

## 2 Material and Methods

### 2.1 Plant material and experimental setup

The dataset used for this study originated from the HTP screening described by Ref. [1]. It includes raw RGB images and defined extracted morphological features from them. Briefly, seeds of *Arabidopsis thaliana* (L.) Heynh. ecotype Col-0 were obtained from the Salk Institute Genomic Analysis Laboratory (http://www.signal.salk.edu/cgi-bin/tdnaexpress). The seeds were surface-sterilised with 70% ethanol and 0.01% Triton X-100, following the protocol described by De Diego et al. [2]. After sterilization, the seeds were transferred to a square plate (120 × 120 mm, P-Lab, Ref. 212358.2) containing half-strength solid Murashige & Skoog (MS) [12] medium (Phytotechlab M519) adjusted to pH 5.7 and supplemented with 0.6% Phytagel, and supplemented with five different priming agents consisting of novel urea derivatives predicted as cytokinin oxidase/dehydrogenase (CKX) inhibitors; 3TFM-2HE, 2OMe-3,5DCl, 2AD5Cl-3Cl, 2AD.5Me-3Cl, or 2AD.5OMe-3,5DCl, as described by Ref. [13]. Each compound was applied at four different concentrations (0.01, 0.1, 1, or 10 μM); untreated seeds served as a control. Three days after germination, the *Arabidopsis* seedlings of similar size were transferred to 48-well plates containing 1 × MS growth medium for control conditions or 1/3 × MS for low-nutrient conditions. The plates were then placed on the OloPhen platform (http://www.plant-phenotyping.org/db_infrastructure#/tool/57), which includes a PlantScreen™ XYZ system (Photon Systems Instruments, PSI, Dr&aacute;sov, Czech Republic) installed in a growth chamber with controlled conditions and equipped with a high-resolution top-view RGB camera that acquires images twice daily [13].

### 2.2 Datasets and plant descriptors

**Fig. 2:**
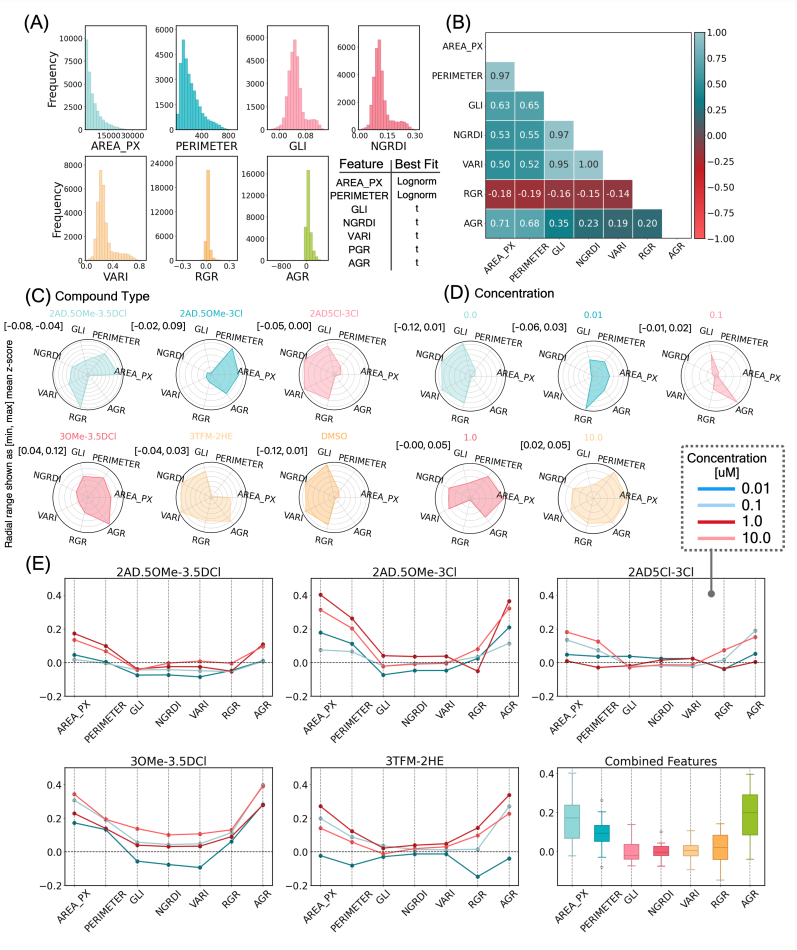
**(A)** Histogram representation of the population variability of each descriptor extracted from high-resolution RGB images of Arabidopsis seedlings primed with five cytokinin analogues (2AD.5Ome-3,5 DCl, 2AD.5Ome-3Cl, 2AD5Cl-3Cl, 3Ome-3,5DCl, or 3TFM-2HE) at four concentrations (0.01, 0.1, 1 or 10 μM). **(B)** Pairwise Pearson correlation coefficients between the seven plant-derived descriptors across all plants and time points. Radar plots of descriptor values z-score normalised (mean=0,σ=1) per descriptor across all plants and time points for representative **(C)** compound and **(D)** concentration. For each radar, the bracketed values above the panel report the radial axis range (min, max) in mean z-score units, to make the scale explicit. **(E)** Parallel plot of descriptor changes expressed as **log_2_ fold changes** relative to the unprimed control, illustrating the magnitude and direction of treatment effects. Descriptor axes are arranged in an arbitrary order; the connecting lines are used solely as a visual aid to follow each treatment profile and do not imply ordinality among descriptors.

Two datasets from the same experimental setup [1] were used to track Arabidopsis growth at early developmental stages. The datasets contain information from a total of 27,761 individual plants imaged for 7 days, organised as a matrix with dimensions (27,761, × 7). The first dataset, referred to as the *Plant-Derived Descriptors Dataset*, comprises quantitative data extracted from red-green-blue (RGB) images after processing using the methodology described by Ref. [13]. It involved plant segmentation and colour indexes calculated through the contribution of the R-G-B channels using custom-developed software that captures seven key descriptors for each plant: projected rosette area (AREAPX) and length of the rosette contour (PERIMETER) in pixels, three colour indexed: Green Leaf Index, capturing relative leaf greenness (GLI), Normalised Green–Red Difference Index, emphasising green tissue relative to red (NGRDI), Visible Atmospherically Resistant Index, a visible-light index robust to illumination changes (VARI), and two relative growth rates: absolute growth rate of the projected rosette area between consecutive imaging days (AGR), and relative growth rate, i.e. AGR normalised by plant size (RGR) [13]. Together, these descriptors provide a detailed quantitative profile of morphological and physiological traits. Their empirical distributions are illustrated in Fig. 2, and formal definitions and units for all seven descriptors are summarised in Supplementary Table S1.

**Fig. 3:**
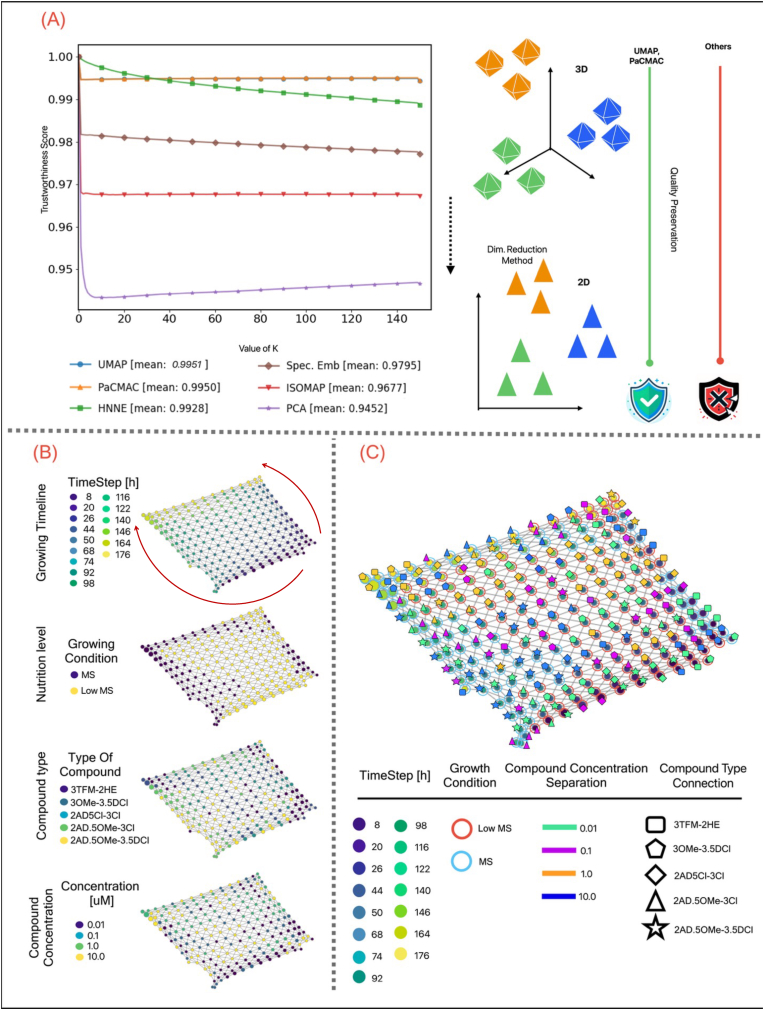
**(A)** Using the tree-structured Parzen estimator, we conducted 200 trials for each method to optimise the trustworthiness score (eq (1)). The graph presents the best solutions. The trustworthiness scores for k values from 0 to 150 were averaged. UMAP emerged as the top performer, achieving an average trustworthiness score of 0.9951, closely followed by PaCMAC (0.9950) and HNNE (0.9928). On the other hand, methods such as spectral embedding, ISOMAP, and PCA performed poorly. These results substantiate our choice of UMAP for inclusion in our analytical pipeline because of its superior performance in maintaining the integrity of neighbourhood relationships in the dataset**. (B)** Visualisation of Euclidean mapper embeddings for the plant phenotyping dataset using the Euclidean mapper. Visualisation of plant growth derived from the RGB image-extracted descriptors, organised based on four external factors: compound concentration, growth timeline, nutrient level, compound type, and **(C)** their interactions. Red arrows indicate the plant growth from early to late developmental stages. Each node in the Mapper graphs in panels (B) and (C) represents a cluster of plants, and edges indicate that two clusters share at least one plant due to overlap between cover regions.

**Fig. 4:**
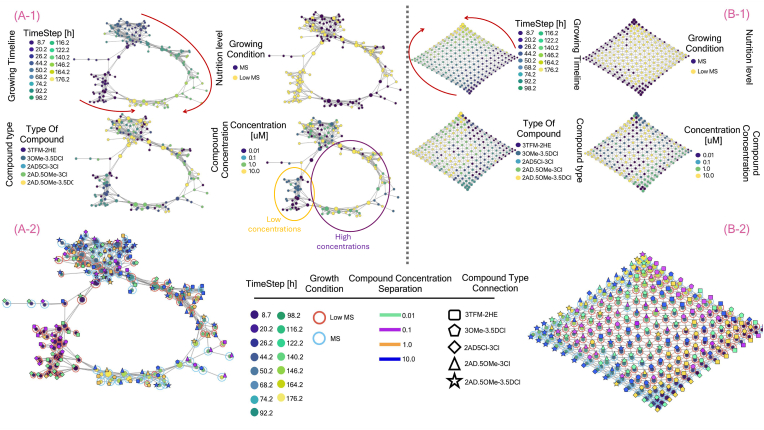
Visualisation of Euclidean mapper embedding for plant phenotyping images. **(A)** and **(B)** show the Euclidean mapper visualisations for the image-based dataset using two distinct dimensionality reduction methods. **(A-1)** shows the results from the SimCLR contrastive learning model, and **(A-2)** presents clusters representing the interaction of factors, while **(B-1)** presents the results from the BYOL contrastive learning model and **(B-2)** the interactions of factors. Both visualisations provide a mapped dataset structure, with each dimensionality reduction method offering a unique perspective on the underlying image data in Euclidean space. Red arrows indicate the plant growth from early to late developmental stages. Yellow and violet ellipses indicated plants grown under low and high compound concentration, respectively.

**Fig. 5:**
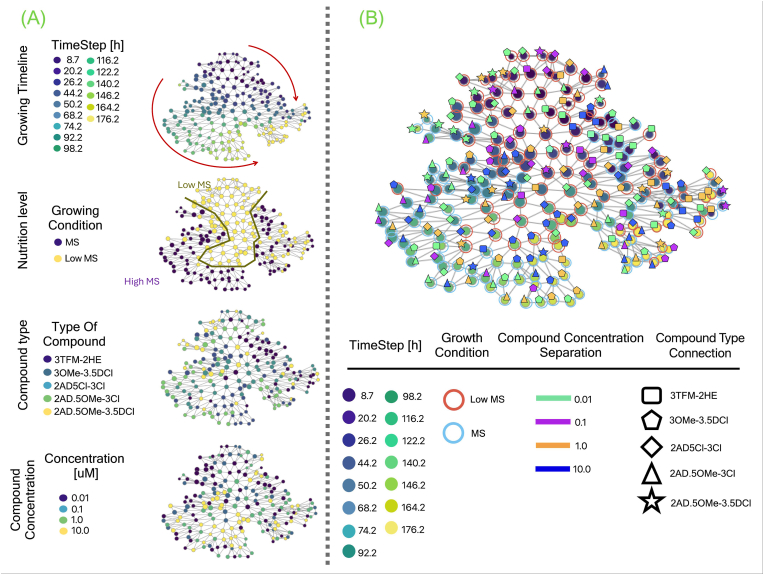
HTDA-Mapper embedding for plant phenotyping quantitative datasets. **(A)** presents the Poincaré mapper visualisation of the dataset based on plant-derived descriptors, influenced by four external factors — compound concentration, growth timeline, nutrition level, and compound type — and **(B)** shows the interactions among them. In these HTDA-Mapper graphs, each node represents a cluster of plants in the hyperbolic embedding, and edges connect clusters that share at least one plant because their cover regions overlap. Red arrows indicate the plant growth from early to late developmental stages. Brown line separated the plants grown under low and normal nutrition.

**Fig. 6:**
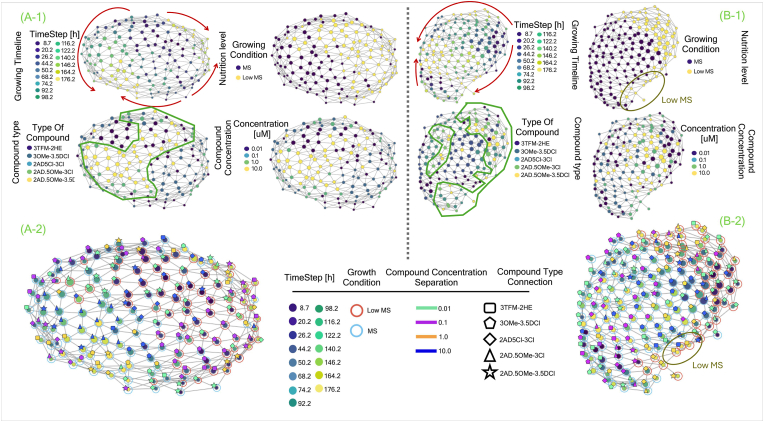
HTDA-Mapper embeddings for plant phenotyping images. This hyperbolic space representation offers an alternative view of data relationships based on the experimental factors. **(A)** and **(B)** present Poincaré mapper visualisations of the image-based dataset, employing two different hyperbolic dimensionality reduction techniques. **(A-1)** uses the SimCLR contrastive learning model, with (**A-2**) as the combinatory representation of all factors, whereas **(B-1)** incorporates the BYOL contrastive learning model, with (**B-2**) as the combinatory representation of all factors. Both models were converted to Poincaré space. Each approach captures unique structural patterns within Poincaré space, revealing the organisation of high-dimensional image data as embedded in hyperbolic geometry. As in Fig. 5, nodes correspond to clusters of plants in Poincaré space, and edges connect clusters that share at least one plant due to overlap in the hyperbolic cover. Red arrows indicate the plant growth from early to late developmental stages. The green polygonal structure grouped plant primed with compounds that induced a similar morphological phenotype. The brown ellipse indicates well-performing plants grown under low nutrition.

**Fig. 7:**
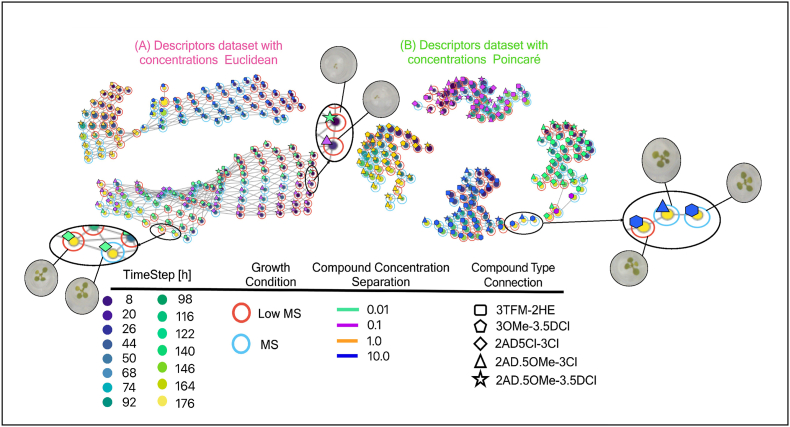
Mapper result visualisations for the dataset of plant-derived descriptors with emphasis on the compound concentration. This figure presents visualisations of the plant growth mapper results derived from RGB-image-extracted descriptors, explicitly highlighting the compound concentration variable, which was identified as a less dominant feature in previous analyses. The dataset, now augmented with this variable, is visualized through the Mapper pipeline in Euclidean **(A)** and Poincaré (**B**) spaces. These visualisations enable focused analysis of patterns in compound concentration within each geometric framework. The black ellipses indicate similar plant growth and morphology among treatments.

Conceptually, this dataset, composed of descriptors extracted from RGB images, can be viewed as a flattened representation of an underlying multifactorial experimental design, in which each plant of each variant was considered for single-plant phenomics. Arabidopsis seedlings were grown *in vitro* under two nutrient regimes (full and low MS), primed with five cytokinin analogues at four concentrations (0.01, 0.1, 1, and 10 μM), and imaged over seven days. Thus, the experiment spans combinations of nutrient regime × compound × concentration × time, with multiple biological replicates per combination. For Mapper analysis, we treat the resulting observations as rows of the (27,761 × 7) descriptors matrix and use the experimental factors as categorical labels to colour and interpret the Mapper graphs (e.g. Fig. 3, Fig. 4, Fig. 5, Fig. 6, Fig. 7).

The second dataset, the *image-based dataset*, comprises raw, high-resolution, top-view RGB images acquired at the OloPhen platform. The initial raw data comprise 704 images, each representing a well plate with 48 individual plants used as independent biological replicates (704 x 48 = 33,792 plants) over 7 days. A rigorous preprocessing pipeline described by Ref. [5], including automated plant detection and validation, was applied to isolate individual plants within each plate. Wells with no germinated plant, severe detection artefacts, overlapping seedlings, or other imaging issues were discarded. Following this procedure, the dataset comprised 27,761 images of individual plants, each represented as an RGB image with dimensions 3 × 128 × 128.

Both datasets are augmented with target variables: time point, growth condition, compound type, and compound concentration. These labels enable an in-depth examination of phenotypic responses across varying experimental conditions, allowing the construction of mapper graphs to model complex trait interdependencies (see Subsections ***2.4 - Euclidean Pipeline*** and ***2.5 - Poincaré Ball Pipeline*** for further details). For each plant and time point, the seven descriptors are extracted from the corresponding segmented RGB image of each Arabidopsis seedling, so that the descriptors and image-based datasets share the same coding that identifies from which plate, well, and day the plant is imaged. They can be treated as paired views of the same experiment.

For the statistical and correlation analysis of the extracted descriptors, we computed pairwise Pearson correlation coefficients across all plants and time points to quantify their linear relationships. For visualisation in the radar plots, descriptor values were z-score normalised (mean=0,σ=1) per descriptor across all plants and time points before plotting, enabling comparison of their relative contributions across different treatments. Radar plots show group-wise mean z-scores with auto scale**.** In the parallel plots, changes in descriptor values are shown as log_2_ fold changes relative to the unprimed control, highlighting the magnitude and direction of treatment effects.

### 2.3 Evaluating dimensionality reduction methods

In data science, particularly when handling complex biological datasets, selecting an appropriate dimensionality reduction (DR) technique is crucial for effective data interpretation and downstream analyses. Dimensionality reduction approaches can be broadly categorised into decomposition-based and manifold learning methods. Decomposition-based methods, such as Principal Component Analysis (PCA) [14], rely on linear projections to capture the directions of maximal variance. In contrast, manifold learning methods preserve nonlinear structures and include techniques such as t-distributed Stochastic Neighbour Embedding (t-SNE) [15], Uniform Manifold Approximation and Projection (UMAP) [16], Isometric Mapping (ISOMAP) [17], Hierarchical 1-Nearest Neighbour Embedding (HNNE) [18], and Pairwise Controlled Manifold Approximation Projection (PaCMAP) [19]. These latter methods have demonstrated utility across diverse datasets, underscoring their capacity to extract meaningful structures and relationships in complex data.

In the context of the approaches described in Section 2.2, dimensionality reduction serves two key purposes. First, Mapper and HTDA-Mapper operate on low-dimensional embeddings that define the cover and clustering, and these embeddings must be amenable to 2D visualisation to inspect treatment- and time-dependent patterns. Second, although the plant-derived descriptors are only seven-dimensional, they exhibit strong correlations and encode multifactor responses, while the image-based features obtained from contrastive learning are high-dimensional. Applying a range of Euclidean and hyperbolic DR methods, therefore, allows us to obtain comparable low-dimensional embeddings for both tabular traits and image features, and to assess how well different approaches preserve local neighbourhood structure and biologically meaningful progression and branching patterns.

Given the critical role of feature reduction in our analyses, we conducted targeted hyperparameter optimisation for a subset of these techniques, adapting each method to maximise performance on our plant-derived descriptor dataset. To systematically evaluate the efficacy of the selected DR methods on the plant dataset, we employed the trustworthiness score as our primary metric [20,21]. Trustworthiness quantitatively measures how well the neighbourhood relationships in high-dimensional space are preserved in a lower-dimensional embedding, a crucial factor for maintaining data integrity after dimensionality reduction.

The trustworthiness score, T(k), is defined by Equation (1):

(Eq. 1) $$T\left(k\right)=1-\frac{2}{nk\left(2n-3k-1\right)}\sum_{i=1}^{n}\sum_{j\in U_{k}\left(i\right)}\left(r\left(i,j\right)-k\right)\text{,}$$

where n represents the total number of points, $U_{k}\left(i\right)$ denotes the set of points that lie in the k-nearest neighbourhood of point i in the low-dimensional embedding but not in the corresponding neighbourhood in the original space, and r(i,j) is the rank of the distance between points i and j in the original high-dimensional space. This metric is instrumental in ensuring that the geometric and relational fidelity of the data is preserved after reduction, a key consideration for complex biological datasets.

In addition to trustworthiness, we computed two complementary neighbourhood-preservation metrics for the plant-derived descriptor data. First, we calculated an average Jaccard neighbourhood overlap between the sets of k-nearest neighbours in the original descriptor space and in each low-dimensional embedding for k=1,…,K, quantifying the agreement between neighbourhood sets in the two spaces. Second, we used a continuity-style neighbourhood recall metric, defined as the average fraction of original k-nearest neighbours that remain among the k-nearest neighbours in the embedding, providing a directional measure of how well local neighbourhoods are retained after dimensionality reduction. The resulting average values for these metrics are reported in **3.1.- *Mapper in Euclidean Space: Traditional Route or Contrastive Learning Integration*** alongside the trustworthiness comparison.

Within the context of our *Euclidean and HTDA-Mapper pipelines*, we selected UMAP as the optimal dimensionality reduction method. The results of our hyperparameter optimisation, including comparative analyses across different techniques and the justification for our final selection, are shown in the Results and Discussion section.

### 2.4 Euclidean pipeline

Our Euclidean pipeline begins with a filter function f:X→R, which reduces the dataset X by mapping data points to a real-number scale. This transformation simplifies the high-dimensional data, preserving essential characteristics while facilitating visual interpretability.

Following this transformation, a *cover function* was employed to segment the output range of f(X)intooverlappingintervals,formingacoverU. The cover is designed with sufficient interval overlap to ensure continuity and capture significant data patterns, providing a cohesive representation of the dataset's structural complexity.

Clustering was then applied to the preimages of these intervals, identifying dense clusters of data points that form the vertices of a simplicial complex K. Edges are then established between vertices that share elements across overlapping intervals, effectively reconstructing the interconnected structure of the data [22].

In applying this Euclidean approach to the *Plant-Derived Descriptor Dataset*, we selected UMAP as the filter function because it minimises the cross-entropy between the distance distributions in the original high-dimensional space and its low-dimensional projection, enabling efficient 2D embedding from our initial 7-dimensional dataset.

We employed a cubical cover approach for the cover function, which is frequently utilised in mapper pipelines. This approach partitions the filtered range into overlapping hypercubes. Specifically, given a partition of the range [a,b] intom intervals with overlap, τ, the cubical cover is defined as${\left\{\left[a_{i},b_{i}\right]\right\}}_{i=1}^{m}\text{,}$ where each$b_{i}-a_{i}=\frac{\left(b-a\right)}{m}\left(1+\tau \right)$. This overlapping approach ensures that key topological features are preserved across intervals.

For clustering, we used Hierarchical Density-Based Spatial Clustering of Applications with Noise (HDBSCAN) [23], a density-based algorithm that does not require a predefined number of clusters. HDBSCAN identifies core samples of high density and expands clusters from these samples, with the stability of each cluster quantified by the following metric (Equation (2)):

(Eq. 2) $$Stability=\sum_{Cluster\;C}\left(\sum_{x\;\epsilon \;C}\max\left(0,{core}_{k}\left(x\right)-\epsilon \right)\right)$$

where ${core}_{k}\left(x\right)$ is the core distance for pointx relative to its k-nearest neighbours andϵ is a threshold for cluster inclusion.

In the resulting Euclidean Mapper graphs (e.g., Fig. 3, Fig. 4, Fig. 7), each node represents a cluster of similar plants rather than an individual plant. Concretely, a node corresponds to the set of samples whose low-dimensional embeddings fall within the same element of the cubical cover and are assigned to the same HDBSCAN cluster. Two nodes are connected by an edge when they share at least one plant, which occurs when clusters from overlapping intervals in the cover have a non-empty intersection. Thus, the Mapper graph aggregates the 27,761 plants into a smaller number of nodes that summarise groups of plants with similar embedding coordinates, while the edge structure reflects shared membership across overlapping regions.

The sensitivity of each component, including UMAP, cubical cover, and HDBSCAN, to their respective hyperparameters necessitated extensive tuning. We utilised the Tree-structured Parzen estimator (TPE) [24] and Density-Based Clustering Validation (DBCV) [25] score to optimise these parameters, ensuring the pipeline's robustness and accuracy. This tuning process significantly increased both the interpretability and visualisation quality of the mapper pipeline, as outlined in Subsection ***2.7.- Hyperparameter Optimisation*** and supported by empirical results in Subsection ***3.1.- Mapper in Euclidean Space: Traditional Route or Contrastive Learning Integration*** section.

For the *Image-Based Dataset*, we adapted a contrastive learning approach to address the challenges posed by the high dimensionality of image data for traditional feature-reduction methods. We employed the SimCLR and BYOL models, which have demonstrated excellent performance across diverse datasets [26]. SimCLR employs a contrastive loss function, focusing on positive example pairs i,j to learn discriminative embeddings. The loss function is defined by Equation (3):

(Eq. 3) $$L_{i,j}=-\log\frac{\exp\left(\frac{sim\left(z_{i},z_{j}\right)}{\tau }\right)}{\sum_{k=1}^{2N}{∥}_{\left[k\neq i\right]}\;\exp\left(\frac{sim\left(z_{i},z_{k}\right)}{\tau }\right)}$$

where sim(u,v) denotes the similarity (e.g. cosine similarity) between two embeddings u and v,τ is a temperature scaling parameter, and N represents the number of positive pairs. This formulation encourages the network to distinguish between similar and dissimilar examples, which is essential for meaningful embedding learning.

In contrast, BYOL operates without using negative pairs. Instead, it aligns the embeddings produced by an online network $f_{\theta }$ with those generated by a target network $f_{\xi }$. The training is governed by a loss function (*L*) that minimises the distance between the projection of an image v by the online network and the projection of an augmented version $v^{\prime }$ by the target network (Equation (4)):

(Eq. 4) $$L={\left\|norm\left(f_{\theta }\left(v\right)\right)-norm\left(f_{\xi }\left(v^{\prime }\right)\right)\right\|}^{2}$$

where norm(·) denotesL2 normalisation. This approach promotes consistency across different views of the same image, leading to more robust feature representations and a deeper understanding of the data's inherent structure. A comprehensive description of the model training and data augmentation processes can be found in subsection ***2.6.- Training Details of Contrastive Learning*** section.

After processing the image dataset to obtain 2D embeddings, we applied the cubical cover and HDBSCAN, both optimised. These advanced methodologies effectively address the complexities of high-dimensional image data, transforming them into actionable insights, as elaborated in subsection ***3.1.- Mapper in Euclidean space: Traditional Route or Contrastive Learning Integration*** section.

### 2.5 Poincaré ball pipeline

Our analysis of the Arabidopsis dataset revealed that while the Euclidean pipeline performs effectively across many datasets, it may overlook subtler hierarchical patterns due to limitations of Euclidean space. We employed the Poincaré ball model, a representation of hyperbolic space characterised by constant negative curvature, to capture these nuances. This space is advantageous for visualising and analysing data with inherent hierarchical structures, such as biological datasets [27]. In our Arabidopsis phenotyping experiment, we treat the experimental factors as a coarse-to-fine hierarchy that reflects how interventions act on the plant. This biologically motivated coarse-to-fine hierarchy of nutrient regime, compound identity, and concentration, introduced above, gives rise to nested and branching relationships in the phenotypic data. Such a multi-level structure is naturally accommodated by hyperbolic space, motivating the use of the Poincaré ball model in the HTDA-Mapper pipeline.

In hyperbolic space, distances increase more rapidly as points move farther from the centre, which is consistent with hierarchical or tree-like data structures, such as those in our datasets. The Poincaré ball model provides an isometric representation that preserves angular relationships, similar to Euclidean space, making it well-suited for our purposes. The distance between two points $x,y\;\epsilon {\;B}^{m}$ in this model is given by Equation (5):

(Eq. 5) $$d_{B}\left(x,y\right)=\cosh^{-1}\left(1+2\;\frac{{∥x-y∥}^{2}}{\left(1-{∥x∥}^{2}\right)\;\left(1-{∥y∥}^{2}\right)}\right)$$

Here, $B^{m}=\{x\in R^{m}:∥x∥<1\}$ denotes the m-dimensional Poincaré ball, and m is the dimension of the hyperbolic embedding space; in this work we use m=2 for the 2D embeddings visualised in our Mapper graphs. These formulations allow us to adapt BYOL and SimCLR contrastive learning models to the Poincaré ball space, optimising them for hyperbolic geometry (please note that all layers and operations need to be adjusted accordingly for the Poincare ball space; see subsection ***2.6.- Training Details of Contrastive Learning***). This adaptation enables the models to capture hierarchical relationships and structures more effectively in the feature space.

In our HTDA-Mapper pipeline for the *plant-derived descriptor dataset*, we modified UMAP to operate in hyperbolic space using the hyperboloid model. In this configuration, the coordinates that span the two space-like dimensions $\text{and}\;{\;x}_{2},\text{while}{\;x}_{0}$ are used for distance calculations. Once the embeddings are learned in the hyperboloid model, we project them into the Poincaré disk a 2D model of the hyperbolic space used for visualisation. This conformal mapping preserves angles and provides a compact view, making it suitable for interpreting the geometric layout. The transformation is straightforward, as indicated in Equation (6):

(Eq. 6) $${disk}_{{\;x}_{1}}=\frac{{\;x}_{1}}{1+{\;x}_{0}},{disk}_{{\;x}_{2}}=\frac{{\;x}_{2}}{1+{\;x}_{0}}$$

These transformations allow UMAP to effectively project embeddings onto the Poincaré ball, preserving the dataset's hierarchical structures.

Traditional cubical cover approaches are not compatible with hyperbolic space because of the unique geometry required for the cover function. We address this by introducing a novel cover function based on a *Riemannian K-means cover* of the Poincaré ball distance metric. This approach segments the embeddings into clusters, with overlap determined by the distances between the centroids and a predefined threshold. The pseudocode is detailed in Algorithm 1 (Supplementary Table S2).

For clustering, the Density-based spatial clustering of applications with noise (DBSCAN) [28] algorithm was adapted to utilise the Poincaré ball distance metric. This metric enables precise clustering in a hyperbolic space while preserving the dataset's hierarchical relationships.

As in the Euclidean pipeline, nodes and edges in the HTDA-Mapper graphs represent clusters and overlaps of phenotypically similar plants rather than individuals. Each node in the Poincaré Mapper graph corresponds to a cluster of samples whose hyperbolic embeddings lie within the same *Riemannian K-means* cell and are assigned to the same DBSCAN cluster. Two nodes are connected by an edge when they share at least one plant, reflecting overlap between cover regions and their associated clusters. Consequently, the number of nodes is much smaller than the number of plants because many plants are aggregated into clusters.

### 2.6 Training details for contrastive learning

The image dataset processed for contrastive learning consists of 27,761 individual *Arabidopsis* seedlings imaged in RGB format (3 × 128 × 128) over time, as introduced in the subsection ***Datasets and Plant Descriptors***. To ensure consistent input scaling, we applied normalisation, a standard practice in computer vision, to stabilise training and enhance algorithm performance.

A key element of training contrastive learning models is generating diverse image views through augmentations. For this purpose, RandomCrop, RandomRotation, and ColorJitter were used to create varied representations of each instance, ensuring that both the SimCLR and BYOL models provide unique perspectives of the same sample. These augmentations are carefully selected to maximise diversity and effectiveness, given the lower resolution of the images, and were optimised specifically for this task through empirical testing.

Both models use a ResNet50 backbone pretrained on ImageNet, chosen for its robustness due to ImageNet's diverse collection. This pretraining approach supports effective feature extraction, an advantage in contrastive learning, where generalisable features are essential for downstream performance. We rely on weights from PyTorch's model library for the Euclidean implementations. The hyperbolic variant of ResNet50 was adapted to the Poincaré ball space using the Hyperbolic Learning Library, enabling operations in hyperbolic geometry. This hyperbolic model is also pretrained based on ImageNet to ensure comparable feature robustness.

The projector head, which is crucial for transforming features into contrastive loss space, consists of a linear layer, batch normalisation, rectified linear unit (ReLU) activation, and a final linear layer to produce the embeddings. We replaced standard Euclidean layers with hyperbolic counterparts for the hyperbolic models to maintain geometric integrity. Transitioning from Euclidean to hyperbolic space requires using hyperbolic equivalents for standard operations, such as the exponential moving average (EMA), which we implement using exponential and logarithmic maps to ensure that feature transformations align with hyperbolic geometry. Notably, the functional form of the contrastive loss functions (Eqs. (Eq. 3), (Eq. 4))) is identical in Euclidean and hyperbolic settings (see Subsections ***2.4.- Euclidean Pipeline*** and ***2.5.- Poincaré Ball Pipeline*** sections); the difference lies in how similarities are computed. For Euclidean models, we use cosine similarity between l2− normalised embeddings in $R^{d}$. For hyperbolic models, embeddings lie on the Poincaré ball and are first mapped to the tangent space at the origin by the logarithmic map; cosine similarity is then evaluated in this tangent space, ensuring that the loss is computed on geometry-aware distances while preserving the usual SimCLR/BYOL objective.

Our training regimen incorporated several strategies to optimise learning. Extended training periods were used to maximise model performance, while early stopping was applied to terminate training when loss reduction plateaued, thereby efficiently managing computational resources. We employed gradient clipping to prevent instability caused by large gradients and an exponential learning rate scheduler to fine-tune the model toward the optimal solution. Mixed precision training further balanced computational efficiency and training effectiveness. For optimisation, adaptive moment estimation with weight decay (AdamW) was used for Euclidean models, whereas Riemannian Adam was applied to hyperbolic models, tailored for optimisation in curved space.

To monitor training progress in this self-supervised setting, we report the contrastive loss, along with top-1 and top-5 instance-retrieval accuracies. Because contrastive learning relies on stochastic data augmentation and random mini-batch sampling, instantaneous accuracy or loss values at a single iteration are not directly comparable across models. Accordingly, we summarise training behaviour using epoch-averaged metrics and learning curves, which provide a more robust and interpretable view of model convergence.

In our self-supervised framework, each mini-batch of size B produces 2B augmented views. For a given anchor view, its positive pair is the second view of the same plant at the same time point, and the remaining 2(B−1) views are treated as negatives. Top-1 accuracy is the proportion of anchors for which the positive view has the highest similarity among all candidates, whereas top-5 accuracy counts anchors where the positive is ranked among the five most similar views.

Training curves report epoch-averaged contrastive loss and mini-batch instance-retrieval accuracies (top-1 and top-5), computed at each epoch across all mini-batches in the training dataset (i.e., averaged over the full training set per epoch) (Supplementary Figs. 1 and 2). This avoids reliance on single iteration values and enables direct comparison of convergence trends across models. The final accuracy and loss values reported in the Results correspond to the mean values from the final epoch after training convergence. These metrics quantify how reliably the model retrieves matching augmented views and do not correspond to supervised classification into biological treatment classes.

After training, the models generate 2D embeddings from the image dataset, which are subsequently analysed within their respective Euclidean and hyperbolic HTDA-Mapper pipelines as shown in ***3.1.- Mapper in Euclidean Space: Traditional Route or Contrastive Learning Integrations*** and ***3.3.- Advancing HTDA-Mapper with Contrastive Learning Techniques.***

In principle, large pretrained vision foundation models could be used as off-the-shelf feature extractors. However, HTP images form a specialised domain in which subtle local changes in morphology and colour are critical, and representations trained only on generic natural-image corpora may not reliably capture these traits. In addition, our HTDA-Mapper pipeline requires embeddings that can be optimised consistently in both Euclidean and hyperbolic geometries with Riemannian operations on the Poincaré ball and adapting extensive pretrained architectures to this setting would require substantial re-engineering beyond the scope of this work.

### 2.7 Hyperparameter optimisation

The Mapper algorithm is a powerful tool for visualising high-dimensional data and identifying underlying patterns. However, it is highly sensitive to parameter selection, resulting in a wide range of possible visualisations. Therefore, hyperparameter optimisation was essential at each step of the mapper pipeline, both for the Euclidean and Poincaré ball approaches. We performed 200 trials for each approach using the TPE optimisation algorithm to improve our visualisation results. The TPE algorithm is particularly suitable for hyperparameter tuning, as it models the objective function probabilistically. This algorithm distinguishes between the most promising hyperparameters and the remaining hyperparameters, allowing it to focus subsequent trials on the best-performing areas of the hyperparameter space. This targeted approach improves the efficiency of identifying optimal hyperparameter configurations.

We used DBCV for scoring during the hyperparameter optimisation process. When labels are unavailable, an objective metric such as the silhouette score is typically used to evaluate clustering results. The silhouette score measures cluster cohesiveness and separation on a scale from −1 to 1. However, this metric is unsuitable for density-based clustering because it ignores noise and relies on distances. In contrast, DBCV is designed for density-based clustering algorithms because it accounts for noise and captures the shape properties of clusters through densities rather than distances. The DBCV score is the weighted average of the validity index for all clusters, as defined below:

(Eq. 7) $$DBCV\left(C\right)=\sum_{i=1}^{\zeta }\left(\frac{\left|C_{i}\right|}{\left|O\right|}\;V_{C}\left(C_{i}\right)\right)$$

In this equation, $C=\left\{C_{i}\right\}$ represents the clustering solution, with 1≤i≤l, indicating the range of clusters. The term $\left|C_{i}\right|$ is the size of the cluster $C_{i}$, |O| is the size of the original dataset, and Vc(Ci) is the validity index for the cluster Ci. The DBCV score ranges from −1 to 1, with higher scores indicating better clustering solutions.

After optimisation, we selected the top five configurations for each approach, highlighting the best configuration and its DBCV score in Supplementary Table S3. The table presents the parameters that were chosen for the visualisations in the Results section. The table includes the mean and standard deviation of the top five results for each approach, along with the best DBCV score achieved during optimisation. We considered the top five results rather than focusing solely on the best score because, although a higher DBCV score generally correlates with better visualisations, the final visualisations were evaluated primarily by plant experts.

## 3 Results and Discussion

In this study, we addressed **three key questions**:

1. Can traditional **Euclidean approaches** reveal meaningful structure and interactions within complex plant phenotyping data?
2. Does embedding data in **hyperbolic space (HTDA-Mapper)** improve our ability to visualise hierarchical and dynamic biological patterns?
3. Can **contrastive learning methods (SimCLR and BYOL)** extract informative features directly from images, reducing the need for manual feature engineering?

To answer these, we analysed over 27,000 *Arabidopsis thaliana* seedlings grown *in vitro* under two nutrient regimes (full and low MS) and treated with five cytokinin-like compounds at four concentrations (0.01–10 μM). Each plate was imaged twice daily for seven days, generating a rich dataset for evaluating Euclidean and hyperbolic pipelines. From each image, seven plant descriptors, Arabidopsis rosette area (AREAPX) and perimeter (PERIMETER) calculated by number of pixels, relative and absolute growth rate (RGR and AGR), and the three colour indexes; GLI, NGRDI, and VARI (Fig. 2 and Supplementary Table S1), were calculated using the software described by Refs. [2,13]. They provide a detailed quantitative profile of the plant morphological and physiological traits under different conditions. As shown in Fig. 2, these descriptors, although closely related (Fig. 2B), contributed to distinct population distribution and profiles across compounds and concentrations, suggesting different mechanisms of action.

### 3.1 Mapper in euclidean space: Traditional route or contrastive learning integration

To find the best method for visualising plant changes over time, without the need for multiple separate representations, we first examined whether standard manifold learning methods could capture meaningful biological variation using the descriptors derived from each plant image. Dimensional reduction (DR) techniques, including PCA, t-SNE, UMAP, ISOMAP, PaCMAP, HNNE, and Spectral Embedding, were compared using the trustworthiness score as a function of neighbourhood size k (Fig. 3A). Across the full range of k values, UMAP and PaCMAP consistently achieved the highest trustworthiness, followed by HNNE and ISOMAP, with Spectral Embedding and, especially, PCA performing less well. As expected, trustworthiness decreased with increasing neighbourhood size *k* for all methods, because preserving larger neighbourhoods is more difficult; methods such as PCA and Spectral Embedding steeply declined, whereas UMAP and PaCMAP maintained high trustworthiness across a wide range of *k*, indicating more robust local-structure preservation. This ranking is supported by the additional neighbourhood-preservation metrics introduced in Subsection ***2.3.- Evaluating Dimensionality Reduction Methods*.** On the plant-derived descriptor data, UMAP and PaCMAP attained the highest average Jaccard neighbourhood overlaps (0.492 and 0.485, respectively) and neighbourhood recall scores (0.634 and 0.613), HNNE and ISOMAP form the next group (Jaccard ≈ 0.287 and 0.295; recall ≈ 0.420 and 0.425), followed by Spectral Embedding (Jaccard 0.268, recall 0.393), with PCA performing worst (Jaccard 0.179, recall 0.281). These concordant patterns across trustworthiness, Jaccard overlap, and neighbourhood recall indicate that UMAP and PaCMAP constitute strong Euclidean baselines for local-structure preservation. Following dimensionality reduction, we employed a cubical cover [6] to segment the data space, facilitating structured cluster mapping.

We used UMAP as the filtering function and HDBSCAN, recognised for its ability to differentiate clusters of varying densities, for clustering, a process essential for the detailed elucidation of plant traits (for a more detailed methodology, see Subsection ***2.4.- Euclidean Pipeline***). The final output is illustrated in Fig. 3B, which displays a 2D mapper graph that clearly represents the plant growth trajectories and nutrient effect (Fig. 3B). The structure of the graphs revealed coherent patterns for nutrient levels and compound application (Fig. 3B and C). However, the representation of plant growth over time is mainly linear (Fig. 3B, red arrows), with the right structures occupied by early developmental stages and the left structures for later stages. Manifold learning has been utilised in scientific research due to its ability to reveal the structure of complex data [29]. It is also emerging as an application in plant science for analysing various data formats, from images to tabular data. Concretely, in plant phenomics, UMAP has been successfully used to predict rice traits using unmanned aerial system imagery [30], suggesting that manifold learning is a promising approach for representing and understanding the complex imagery of plants grown under multifactorial stress conditions.

Next, we tested whether image-based embeddings trained via contrastive learning could replace manually extracted descriptors. We used the raw RGB images from a 48-well plate setup of *Arabidopsis thaliana.* We applied the Euclidean Mapper approach with contrastive learning, as outlined in Fig. 1B. From the total of 704 plates with 48 plants each imaged for seven days, we isolated the individual plants and standardised them to an RGB format of 3 x 128 x 128, resulting in a robust dataset structure of 27,761 x (3 x 128 x 128). Then, SimCLR and BYOL models were trained on 27,761 standardised images of individual plants for 7 days (Fig. 4). The Euclidean SimCLR model achieved a training loss of 4.145, top-5 accuracy of 81.03%, and top-1 accuracy of 69.8%. These values correspond to epoch-averaged metrics (mean over the final epoch; Supplementary Fig. S1–S2). The reported top-1 and top-5 values correspond to the instance-retrieval task defined in Section 2.6***.- Training Details for Contrastive Learning*** and therefore quantify how reliably the model matches augmented views of the same plant among all views in a mini-batch.

In contrast, the Euclidean BYOL model yielded training losses of 0.0685, top-5 accuracy of 80.7%, top-1 accuracy of 68.5% (mean values over the final epoch; Supplementary Fig. S2), and higher coherence in the 2D representation than SimCLR (Fig. 4A and B), making it easier to distinguish the influence of different factors on plant growth, and showing a very similar representation to that obtained from the descriptors (Fig. 3B and C). However, both continued to represent time linearly (Fig. 4, red arrows), and even concentrations when used with the SimCLR contrastive learning model (Figs. 4A–1, yellow and violet ellipses). In practice, this means that contrastive learning effectively replaces the manual feature-extraction pipeline: the Mapper graphs built from BYOL-derived image embeddings recover the same treatment structure as those constructed from the extracted descriptors, while additionally capturing image-level nuances without the need for post hoc image analysis. However, in both designed pipelines (Fig. 3, Fig. 4), we observed that UMAP in Euclidean space failed to capture the temporal diversity of plant growth, resulting in a linear appearance (Fig. 3, Fig. 4). In this regard, a recent study by Ref. [27] revealed that UMAP performed poorly in representing lettuce growth, primarily due to a time bias.

### 3.2 HTDA-mapper: A new perspective

To solve the limitation of the linear representation employed by the Euclidean mapper, we implemented HTDA-Mapper, embedding the same dataset composed by descriptors with the Poincaré ball space (Fig. 5). By integrating the HTDA-Mapper pipeline, we employed a modified UMAP that projects the data directly into the Poincaré ball space, improving the adaptability of our analysis to nonlinear representations in hyperbolic space to reflect better the complex relationships in biological process. This innovative approach includes a novel cover function (detailed in Algorithm 1, Supplementary Table S2), specifically designed to navigate the curvature of hyperbolic space effectively. Additionally, we employed hyperbolic DBSCAN adapted for Poincaré distances (Eq. (6)) to generate Mapper graphs that provide richer, more accurate representations of the data.

Using this hyperbolic approach, we revealed a structure that was difficult to resolve in Euclidean space (Fig. 5). In the HTDA-Mapper graphs based on plant-derived descriptors, plants first organise into coarse branches corresponding to the two nutrient regimes, and these branches split into sub-branches that reflect specific priming compounds and concentration levels over time. Along each branch, the seven-day growth timeline is visible as an ordered progression of nodes, while at the tips of branches we observe more specialised phenotypic states associated with particular compound–dose combinations. This nested organisation of nutrient regime, compound, concentration, and time is what we refer to as a hierarchical pattern in this context, and it is naturally represented as branching trajectories in hyperbolic space. Some observed results: plants growth with different nutrition mainly clustered together but some nodes from low nutrition appeared closer to high nutrition, suggesting plants performing better under limited nutrient availability (Fig. 5A). Similarly, primed with compound 3TFM-2HE (dark blue nodes) clustered closely together, whereas those primed with 2AD.5OMe-3Cl (yellow nodes) showed wider dispersion, suggesting a variable response, most probably due to concentration effect that also showed very disperse distribution (Fig. 5). Moreover, combining all factors revealed concentration-dependent clustering (Fig. 5B). Plants grown under low nutrients and primed with low compound concentrations were clustered in the upper part of the map, whereas those under full and low nutrients and primed with the compounds at high concentrations appeared in the lower sections, indicating dose-specific effects consistent with biological observations, with specific concentrations being more effective in improving growth under limited nutrient conditions, as observed by Ref. [1]. These findings underscore the potential of this innovative HTDA-mapper-based representation model for improved treatment separation and, hence, classification. This approach highlights variability across plants and treatments, making it valuable for integration into chemical high-throughput screening pipelines.

### 3.3 Advancing HTDA-mapper with contrastive learning techniques

Finally, we adapted the contrastive learning models SimCLR and BYOL to operate in hyperbolic geometry. This modification allowed the pipeline to process not only quantitative datasets but also to analyse image data directly. The changes also included utilising our novel hyperbolic cover function and a hyperbolically adapted DBSCAN for clustering. We used the same image dataset of 27,761 standardised RGB images of *Arabidopsis* seedlings grown under multifactorial conditions. The hyperbolic SimCLR model achieved a training loss of 2.9874, a top-5 accuracy of 83.01%, and a top-1 accuracy of 71.77%, outperforming its Euclidean counterpart. All reported loss and top-1/top-5 values are epoch-averaged and taken as the mean over the final epoch (see Supplementary Figs. S1 and S2 for the full training trajectories). These values are obtained under the same contrastive loss formulation as in the Euclidean case, where the loss has identical functional form in Euclidean and hyperbolic spaces and, in the latter, similarities are evaluated in the tangent space at the origin via the logarithmic map. Hyperbolic BYOL models reduced training losses to 0.0542, producing smooth, interpretable embedding.

The resulting 2D HTDA-Mapper visualisations highlighted clear progression and branching patterns in the image-based embeddings, reflecting non-linear growth responses (Fig. 6). Growth timelines do not form a single straight gradient but instead follow curved trajectories whose shapes depend on nutrient levels: under limited nutrient conditions, some plants cannot grow adequately and end the experiment as smaller, poorer-performing rosettes, whereas under full nutrition, trajectories extend towards larger, more vigorous plants. For SimCLR, the Mapper graph showed two diverging time-associated trends, with later developmental stages tending to appear towards the periphery (left/right extremes) (Fig. 6A, red arrows). This arrangement partially aligned with the nutrient regime, with low-nutrient plants concentrated towards the centre/right region and full-nutrient plants towards the left (Fig. 6A). By contrast, the BYOL-based representation displayed an inverted temporal organisation, with earlier stages more frequent in peripheral nodes and later stages clustering closer to the centre (Fig. 6B, red arrows). Nutrient effects were broadly consistent between models; however, BYOL revealed a distinct low-nutrient region associated with better-performing plants. This interpretation is supported by the combined-factor colouring, where the lower part of the Mapper is enriched in plants treated with high concentrations (10-1 μM) of 3TFM-2HE (Fig. 6B, black ellipse). The convergence of high-concentration treatments across nutrient levels suggests potential roles in nutrient-use efficiency, aligning with biological expectations [1]. These results underscore the effectiveness of our approach for biological studies utilising optical sensors. Moreover, this approach enables the biological effects of closely related compounds to be distinguished, grouping them together when they induce similar plant growth responses (Fig. 6).

### 3.4 Unveiling the subtleties: A closer look at a lesser dominant feature

We further examined concentration as a single dominant factor to validate the model's sensitivity (Fig. 7). We compared both pipelines to validate the previously obtained results. Both the Euclidean space and the HTDA-Mapper pipeline provided effective separation of the compounds and concentration levels (Fig. 7). However, in Euclidean space, even when the pipeline was able to group similar plants (e.g., 2AD.5OMe-3Cl at the lowest concentration under normal and low nutrition), the data were organised into two submaps, mainly defined by time, which goes from right (earlier age) to left (later age) (Fig. 7A). In contrast, in HTDA-Mapper, more precise separation was achieved. Furthermore, as above, the Euclidean mapper did not adequately represent temporal progression, instead producing a clear linear pattern.

Interestingly, at the 10 μM concentration, plants primed with 3OMe-3.5DCl under low-nutrient conditions were grouped with those primed with the same compound under optimal conditions, and clustered closer to those treated with 2AD.5OMe-3Cl, presenting a similar phenotype, highlighting these compounds as possible effective plant growth promotors and stress alleviators (Fig. 7B). Taken together, our pipeline using HTDA-Mapper and contrastive learning was sensitive enough to detect subtle concentration-dependent effects and similarities between compounds, even when they are chemically closely related. These findings are promising for uncovering both similar and distinct responses across chemical libraries or biostimulants and for identifying effective concentrations for plants grown under complex growth conditions, particularly in large datasets from high-throughput screening approaches.

## 4 Conclusion

In the omics era, the representation and interpretation of high-dimensional datasets remain a challenge for researchers. One clear example is plant phenomics, a rapidly growing field that uses advanced imaging and sensor technologies to capture dynamic changes across plant developmental stages and environmental conditions. These technologies generate a massive amount of data, often in the form of images or multidimensional quantitative descriptors. However, analysing and interpreting this information with traditional statistical and visualisation tools remains time-consuming and limited, as conventional methods struggle to preserve the nonlinear, hierarchical, and temporal structure inherent to biological systems.

Here, we present a transformative approach to overcoming these challenges. Our study demonstrates that topological manifold learning, particularly hyperbolic topological data analysis (HTDA), offers a robust framework for clustering and visualising complex, high-dimensional datasets in multiple formats, including quantitative omic data and images. Unlike traditional PCA, the use of the Euclidean distance within UMAP space [31,32] yields superior performance because of its refined approach to analysing quantitative datasets. This method has recently gained attention as a valuable tool for enhanced visualisation and interpretation of biological data, including high-dimensional transcriptomic datasets [33,34]. Although Euclidean UMAP can effectively summarise large plant datasets, it remains limited by the intrinsic linearity of the underlying space.

Integrating contrastive learning into Euclidean space enhances representation by enabling the direct extraction of patterns from non-quantitative data, such as images, without manual labelling or handcrafted feature extraction (Fig. 4). This advancement broadens the potential applications within TDA. However, despite these improvements, we observed that the resulting Mapper struggled to efficiently represent plant phenotyping data, primarily due to temporal variations in individual phenotypic changes (time bias) [27], likely because of the inherent linear representation of Euclidean space.

To address this issue, hyperbolic space was combined with the Poincaré ball mapper within our methodology. This integration represents more than a mere incremental improvement [22] in our capacity to discern and interpret complex biological data patterns. By exploring the relatively uncharted domain of hyperbolic geometry within TDA, our work highlights the enhanced analytical ability of HTDA-Mapper in capturing the complex phenotypic expressions of plants influenced by diverse environmental and treatment interactions. This approach enables more effective cluster analysis by projecting the data into a hyperbolic space, optimising data separability, and revealing hidden patterns that linear representations, such as Euclidean space, fail to capture, including diverse Arabidopsis phenotypic trajectories over time.

Beyond these methodological advances, HTDA-Mapper also provided biological insights that were not apparent with conventional Euclidean approaches. In Euclidean space, concentration effects and compound responses often appeared partially intermixed, and temporal progression tended to collapse into nearly linear trends. By contrast, the hyperbolic maps revealed branching growth trajectories and more precise separation of treatment responses. For example, in the plant-derived descriptor space, HTDA-Mapper differentiated low-nutrient plants treated with low compound concentrations from those treated with high concentrations, highlighting a dose-dependent improvement in growth under limiting nutrients. Similarly, in the image-based hyperbolic embeddings, plants treated with 10 μM compounds formed distinct, central clusters, whereas plants treated with intermediate concentrations formed peripheral branches, mirroring non-linear dose–response relationships. Finally, HTDA-Mapper grouped plants treated with 3OMe-3,5DCl and 2AD.5OMe-3Cl under both low and optimal nutrient conditions, suggesting shared modes of action and identifying them as promising candidates for further evaluation as growth promoters and stress alleviators. Together, these examples illustrate how hyperbolic topology can uncover subtle, biologically meaningful patterns that remain obscured in traditional Euclidean visualisations.

In summary, we introduce a robust, geometry-aware pipeline for analysing high-dimensional biological data, from quantitative measurements to raw images, that more faithfully reflects the complexity of biological processes. By integrating topological data analysis, hyperbolic geometry, and contrastive learning, this methodology can revolutionise how researchers handle large-scale data from plant phenomics and other omics disciplines. Beyond its application to *Arabidopsis*, HTDA-Mapper holds potential for diverse datasets, enabling the discovery of novel genotypes, compounds, genes, and hidden traits. This framework paves the way towards a new generation of AI-driven, interpretable, and unsupervised data analysis tools for understanding multifactorial biological interactions that shape plant growth, performance, and resilience.

## Authors contribution

J.Z., N.D.D., L.K., L.S., and V.S. conceived the manuscript's structure. J.Z. and N.D.D. led the writing of the manuscript and experimental work. L.S. and N.D.D. contributed to the plant phenotyping experiments. J.Z. and L.K. contributed to the experimental work regarding the machine learning models. J.Z., N.D.D., L.K., L.S., and V.S. contributed to the writing of the manuscript. All authors reviewed and agreed on the manuscript before submission.

## Funding

This work was supported by the project Interdisciplinary approaches for the development and application of new materials in industrial, agricultural and medical practice, reg. no. CZ.02.01.01/00/23_021/0008909 of the ERDF Programme Johannes Amos Comenius.

## Declaration of competing interest

The authors declare that they have no known competing financial interests or personal relationships that could have appeared to influence the work reported in this paper.

## Data Availability

Upon acceptance, the codes and all material used in this research will be freely available at HYPERLINK: https://github.com/JZdrazilX/MML and data at ZENODO: 10.5281/zenodo.17952279.
