# Supplementary material for: Hyperbolic topological data analysis mapper reveals dynamic trait–environment patterns in plant phenomics
Source: Plant Phenomics. 2026 Feb 27;8(2):100186. doi: 10.1016/j.plaphe.2026.100186 (PMC13316264; doi:10.1016/j.plaphe.2026.100186)
Supplement: Multimedia component 1 [file mmc1.docx]

**Supplementary Table S1**. Description of the morphological descriptors extracted from the Arabidopsis RGB images.

| **Descriptor** | **Full name** | **Definition / biological meaning** | **Computation / notes** |
| --- | --- | --- | --- |
| AREAPX | Projected rosette area | Projected area of the rosette in the segmented RGB image, representing overall rosette size. | Number of pixels classified as belonging to the rosette region in the plant mask for a given imaging day. |
| PERIMETER | Rosette perimeter | Length of the rosette contour in the segmented image, reflecting rosette shape and boundary complexity. | Perimeter of the connected rosette mask, computed from the plant segmentation. |
| GLI | Green Leaf Index | Colour index capturing the relative dominance of green over red and blue channels, related to leaf greenness. | $GLI= \frac{2G-R-B}{2G+R+B}$where R, G, B are mean red, green, and blue channel intensities of the rosette. |
| NGRDI | Normalised Green–Red Difference Index | Index emphasising green tissue relative to red, sensitive to chlorophyll content and leaf health. | $NGRDI=\frac{G-R}{G+R}$, with R and G the mean red and green channel intensities of the rosette. |
| VARI | Visible Atmospherically Resistant Index | Visible-light vegetation index robust to illumination variation, describing relative greenness in the visible spectrum. | $VARI=\frac{G-R}{G+R-B}$ using mean rosette R, G, B intensities. |
| AGR | Absolute growth rate | Absolute increase in rosette area between consecutive imaging days, quantifying daily rosette expansion. | $AGR\left( t \right)=AREAPX\left( t+1 \right)-AREAPX\left( t \right),$  $where t and t+1 are consecutive$ |
| RGR | Relative growth rate | Growth rate normalised by rosette size, capturing proportional daily increase in area. | $RGR\left( t \right)= \frac{AGR(t)}{AREAPX(t)}$ using AGR as defined above. |

**Supplementary Table S2**. Algorithm 1.

| **Algorithm 1.-** K-Means Cover in Hyperbolic Space | |
| --- | --- |
| 1:  2:  3:  4:  5:  6:  7:  8:  9:  10:  11:  12:  13:  14:  15:  16:  17:  18:  19:  20:  21:  22:  23:  24:  25:  26:  27:  28:  29:  30:  31:  32:  33: | ***Require:*** $\boldsymbol{X}$*: Dataset in the Poincaré ball space, n clusters: number of clusters,*  *overlap threshold: overlap criterion*  **Initialize:**  Set manifold $M$ to the Poincaré ball  Use distance metric $d$ defined by Eq. 5  **Execute Riemannian K-means:**  Initialize centroids using 'kmeans++' adapted for hyperbolic space if there are no precomputed centers  **while** not converged do  **for** each point $x_{i} \in X$ **do**  Assign $x_{i} to the nearest centroid c_{j}\mathrm{minimizing}d\left( x_{i}, c_{j} \right)$  **end for**  **for** each centroid $c_{j}$ **do**  Recompute $c_{j} as the mean of all points assigned to c_{j}\mathrm{using}d$  **end for**  **end while**  **Store results:**  cluster centers ← centroids from K-means  **for** each point $x_{i}\in X, each centroid c_{j}$ **do**  cluster distances $\left[ i,j \right] \leftarrow d\left( x_{i}, c_{j} \right)$  **end for**  **for** each pair of centroids $\left( c_{i},c_{j} \right)$ **do**  centroid distance $\left[ i,j \right] \leftarrow d\left( c_{i},c_{j} \right)$  **end for**  **Determine cover:**  **for** each point $x_{i}\epsilon X$ **do**  Identify nearest centroid $c_{j}^{*}\mathrm{minimizing}d\left( x_{i},c_{j} \right)$  Set cover matrix $\left[ i, j^{*} \right]$ ←True  **for** each centroid $c_{j}$ **do**  Compute$r=d\left( x_{i},c_{j} \right)/ d\left( x_{i},c_{j}^{*} \right)$  if $r \leq1+$ + overlap threshold **then**  Set cover matrix $\left[ i, j \right]$ ←True  **end if**  **end for**  **end for**  return **cover matrix** |

**Supplementary Table S3.** Hyperparameter optimization results

| **Method** | **Chosen Hyperparameters** | | **Mean** | **Std. Dev.** | **Best DBCV Score** |
| --- | --- | --- | --- | --- | --- |
| **Desc. Euc.** | n_neighbors = 303,  metric = cosine,  min_dist = 0.459,  spread = 0.650,  n_intervals = 15,  overlap_frac = 0.392, min_cluster_size = 154, cluster_method = leaf, | -0.545 | | 0.0143 | -0.524 |
| **Desc. Hyp.** | n_neighbors = 136,  min_dist = 0.425,  spread = 0.620,  n_clusters = 154,  overlap_frac = 0.603,  eps = 1.978,  min_samples = 160, | -0.471 | | 0.0069 | -0.460 |
| **Image.Euc.SimCLR** | n_intervals = 9,  overlap_frac=0.480, min_cluster_size=48, cluster_method = eom, | -0.683 | | 0.0019 | -0.680 |
| **Image. Euc. BYOL** | n_intervals = 15,  overlap_frac=0.348, min_cluster_size=48, cluster_method = eom, | -0.574 | | 0.0001 | -0.574 |
| **Image.Hyp.SimCLR** | n_clusters = 154,  overlap_frac = 0.361,  eps = 0.152,  min_samples = 200, | -0.681 | | 0.0011 | -0.679 |
| **Image. Hyp. BYOL** | n_clusters = 154,  overlap_frac = 0.361,  eps = 0.111,  min_samples = 76, | -0.679 | | 0.0008 | -0.677 |
