# Supplementary material for: Hyperbolic topological data analysis mapper reveals dynamic trait–environment patterns in plant phenomics
Source: Plant Phenomics. 2026 Feb 27;8(2):100186. doi: 10.1016/j.plaphe.2026.100186 (PMC13316264; doi:10.1016/j.plaphe.2026.100186)
Supplement: Multimedia component 2 [file mmc2.docx]

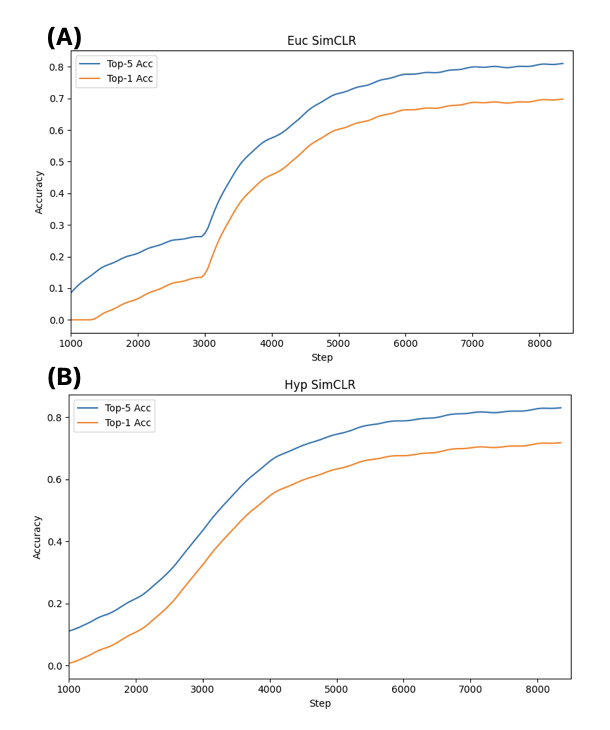


Figure S1. Training curves of top-1 and top-5 instance-retrieval accuracy (mini-batch evaluation) as a function of optimisation step for (A) Euclidean SimCLR and (B) hyperbolic SimCLR. Final (converged) retrieval accuracies reported in the main text: Euclidean SimCLR **top-1 = 69.8%**, **top-5 = 81.03%**; hyperbolic SimCLR **top-1 = 71.77%**, **top-5 = 83.01%**.


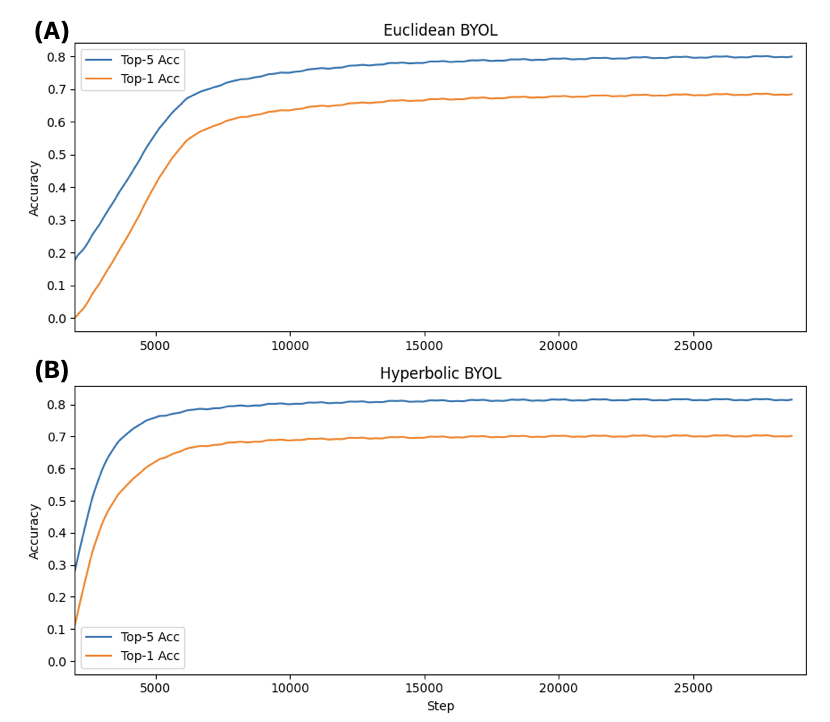


Figure S2. Training curves of top-1 and top-5 instance-retrieval accuracy (mini-batch evaluation) as a function of optimisation step for (A) Euclidean BYOL and (B) hyperbolic BYOL. Final (converged) retrieval accuracies reported in the main text: Euclidean BYOL **top-1 = 68.5%**, **top-5 = 80.7%**; hyperbolic BYOL **top-1 = 70.2%**, **top-5 = 81.5%**.
